# Methods for Improving Image Quality and Reducing Data Load of NIR Hyperspectral Images

**DOI:** 10.3390/s8053287

**Published:** 2008-05-19

**Authors:** Ferenc Firtha, András Fekete, Tímea Kaszab, Bíborka Gillay, Médea Nogula-Nagy, Zoltán Kovács, David B. Kantor

**Affiliations:** Corvinus University of Budapest, Faculty of Food Science, Department of Physics and Control, Somlói út 14-16, H-1118 Budapest, Hungary

**Keywords:** Hyperspectral, noise, data-extraction, carrot, moisture-content

## Abstract

Near Infrared Hyperspectral Imaging (NIRHSI) is an emerging technology platform that integrates conventional imaging and spectroscopy to attain both spatial and spectral information from an object. Two important problems in NIRHSI are those of data load and unserviceable pixels in the NIR sensor. Hyperspectral imaging experiments generate large amounts of data (typically > 50 MB per image), which tend to overwhelm the memory capacity of conventional computer systems. This inhibits the utilisation of NIRHSI for routine online industrial application. In general, approximately 1% of pixels in NIR detectors are unserviceable or ‘dead’, containing no useful information. While this percentage of pixels is insignificant for single wavelength imaging, the problem is amplified in NIRHSI, where > 100 wavelength images are typically acquired. This paper describes an approach for reducing the data load of hyperspectral experiments by using sample-specific vector-to-scalar operators for real time feature extraction and a systematic procedure for compensating for ‘dead’ pixels in the NIR sensor. The feasibility of this approach was tested for prediction of moisture content in carrot tissue.

## 1. Introduction

Non-destructive, non-contact and fast measurement methods are in great demand for on-line industrial quality control tasks. Optical methods, like machine vision systems, allow real-time classification or discrimination of objects on the processing line. By processing the spatial distribution of its RGB coordinates, an object can be identified, its spatial location can be determined and its visible properties, such as colour and shape, can be described by quantitative properties [1-3]. Spectroscopy is another optical method used for routine quality analysis in industry. Spectral properties, such as NIR reflectance, can detect invisible features, e.g. existence of chemical components on the surface. In order to detect a certain feature on a particular object, characteristic wavelengths must be determined by the analysis of sample spectra from that object [4]. Features of interest can be detected by measuring the reflectance on the characteristic wavelengths determined by this analysis. Multi-spectral imaging, much like RGB imaging, can measure the spatial distribution of reflectance at numerous wavelengths (typically ≤ 10). This fast and non-contact measurement method can be also used for real-time controlling or quality control tasks [5, 6].

**Figure 1.**
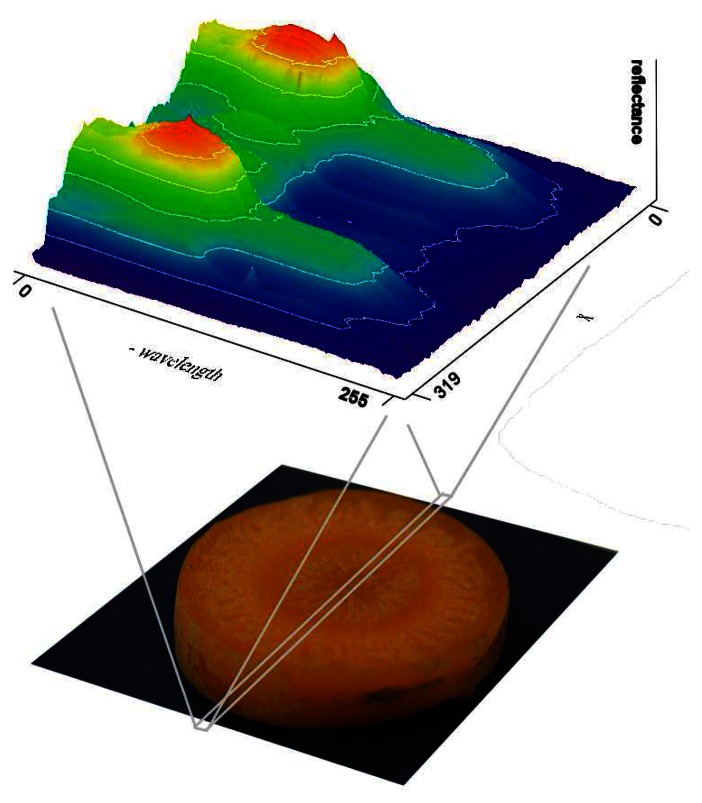
Push-broom measurement setup.

Hyperspectral imaging extends the concept of multispectral imaging to the measurement of images at hundreds of contiguous wavelengths. This non-destructive, non-contact technology was first used for airborne remote sensing applications [7], and since then it has been demonstrated as feasible for many quality control applications in the food and pharmaceutical industries [8]. A number of configurations exist for acquiring hyperspectral images, including the “push-broom” setup [9] and the variable filter focal-plane array [10]. In the push-broom setup, a spectrograph disperses light reflected from a line segment of a sample into a spectrum, mapping all points of the examined line into a rectangular area of a sensor matrix (Figure 1).

The sensor scans this rectangular area with given spatial, spectral and signal resolution, grabbing an Intensity(X,W) matrix, where X is the spatial axis and W represents the spectral axis. Moving steadily perpendicular to the examined line in the Y direction and performing a line scan at each spatial position, the spectra of each pixel can be measured on the surface. The result is an Intensity(X,Y,W) matrix, commonly called a “data hypercube”. This push-broom method can be used in preliminary experiments for determining characteristic wavelengths of a certain property for a given sample type.

Spectra from hyperspectral experiments are generally rather noisier than those obtained in instrumental spectroscopy. This is due to a number of factors: in NIRHSI the sample surface is not isolated, so the illumination is not homogeneous; the unevenness of the sample surface causes high intensity variance; the sensitivity of the system is not homogeneous, and some pixels of the sensor- matrix can be unserviceable. Moreover, the outputs of hyperspectral measurements (hypercubes) are of enormous size, typically in the order of mega- or giga-bytes, which poses significant problems for data storage and processing. This array of data can be analysed by robust mathematical or statistical methods to extract significant features, however, such analysis tends to be time consuming, given the large size of the datasets to be analysed.

In this paper, a strategy for compensation of unserviceable pixels in the NIR detector is presented, and an approach for the reduction of hyperspectral data by real-time extraction of examined features using vector-to-scalar operators is discussed. As an application of the approaches presented, the NIRHSI properties of carrot have been investigated for estimation of the moisture decrease during drying. Preliminary experiments were performed to determine the suitable data reduction operator (target function) for extraction of the moisture content feature. Image processing and statistical algorithms were used to analyse the data in order to determine the optimal target function.

## 2. Hyperspectral imaging system

**Figure 2.**
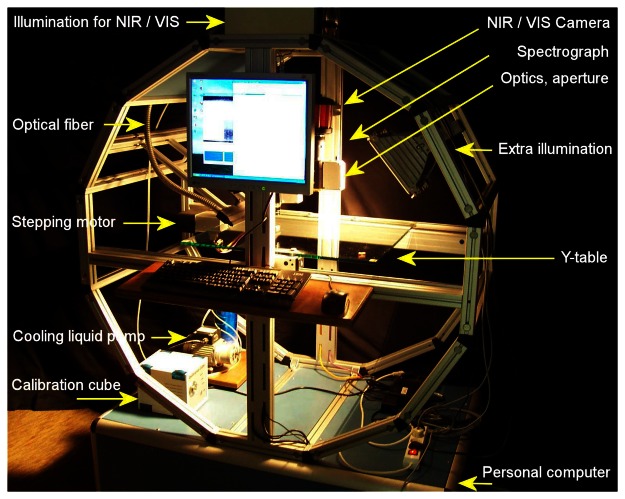
Hyperspectral imaging system.

The hyperspectral imaging system employed in this research (Figure 2) allowed two different configurations: one for imaging in the visible-very NIR range (400 – 1000 nm) and another for imaging in the NIR range (900 – 1700 nm).

The NIR configuration was used in this study. The system consists of a linear translation table, illumination source (DC regulated light feedback fibre optic, Model 3900, http://www.illuminationtech.com), objective lens (2/3″ C-mount broadband coated lenses, Schneider–Kreuznach CINEGON), Specim N17E spectrograph (Spectral Imaging Ltd., Oulu, Finland) operating in the wavelength range of 900 – 1700 nm, detector (LuxNIR camera with InGaAs focal plane array, effective resolution of 320 × 256 pixels by 12 bits, 30×30 μm pixel pitch, 98% pixel operability) and PC. The linear translation table was driven by Isel LF4 mechanics and Isel TMO-4403 (PICMIC) stepping motor that could be controlled by textual commands via an RS-485 interface (http://www.isel.com). Wavelength calibration was performed using Mercury-Cadmium and Helium lamps in NIR and visible ranges and the heat extracted from the NIR camera sensor by a Peltier cooler was removed by an external liquid pump.

Reflectance calibration was required to account for non-homogeneous spectral response of detector. The relative absorbance value (‘RA’) was calculated from the measured signal (‘x’) as shown in equation 1, where the dark field reading (‘min’) was obtained by covering the optics with a lens cap and the bright field reading (‘max’) was obtained by measuring the reflectance of a gold-covered plate. Reflectance (‘R’) and absorbance (‘A’) are defined in equations 2 and 3.

(1) $$RA=\frac{\max-x}{\max-\min}.4095$$

(2) $$R=1-\frac{RA}{4095}$$

(3) $$A=\mathrm{lg}\left(\frac{4095}{4095-RA}\right)$$

## 3. Methodology

### 3.1. Unserviceable NIR sensor pixels

**Figure 3.**
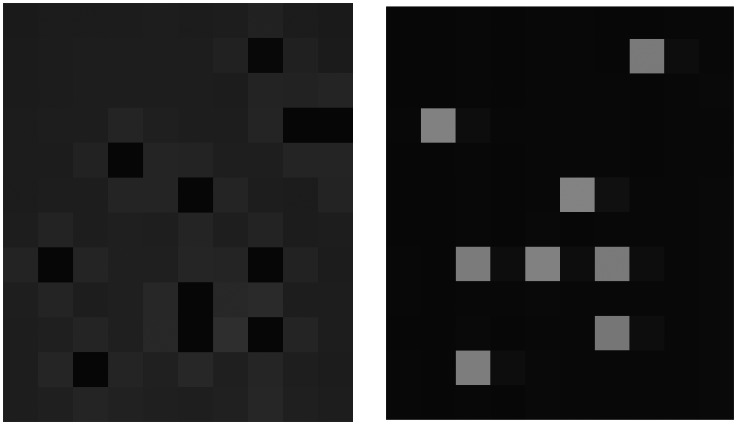
Dark (a) and bright (b) pixel's effect and superposition.

Two types of unserviceable pixels observed for the NIR sensor (InGaAs focal plane array) are described below:

- Extraordinarily dark pixels: These pixels behave like a stone dropped into water, resulting in a slightly higher intensity level for their four neighbours [Figure 3 (a)]. The superposition effects were additive, therefore one fourth of the missing signal of dark pixels had to be subtracted from that of the four neighbours.
- Extraordinarily bright pixels: These pixels resulted in a “light shadow” on the pixel directly to their right; this “light shadow” also affected far neighbours with exponentially decreasing intensity [Figure 3(b)].

Standard noise removal algorithms for image processing are not applicable to the problems listed above. Therefore, the following steps are proposed to deliver suitably homogeneous frames:

#### A. Identification of extraordinarily dark and bright pixels

Dark and bright pixels have to be identified on a frame of a grey (mid-intensity level) surface when calibrating extraordinary pixels. Firstly, the intensity variance for a square area of a given size (e.g. 10×10 pixels) on the grey surface is calculated. Then this variance is multiplied by a number, N (e.g. 4), to define a threshold value. Pixels with intensity values above this threshold are classified as extraordinarily bright, while pixels with intensity values below this threshold are classified as extraordinarily dark.

#### B. Correction of extraordinarily dark and bright pixels

**Figure 4.**
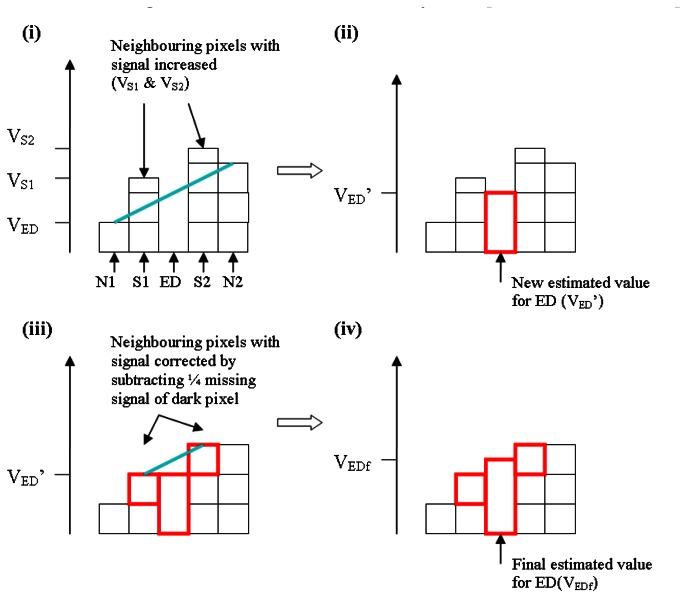
Schematic showing correction of extraordinarily dark pixel based on interpolation.

The steps required for correction of an extraordinarily dark pixel (ED) by interpolation in shown in Figure 4. Surrounding the ED are 2 bright shadow pixels (S1, S2) which are neighboured by two normal pixels (N1, N2); firstly, a linear regression is made between N1, N2 to estimate the value of ED [Figures 4(i) & (ii)]. S1 and S2 are then corrected by subtracting ¼ of the error (i.e. difference between actual and estimated values) of ED [Figure 4 (iii) ]. Finally, a linear regression is made between the corrected values of S1 and S2 to re-estimate the value of ED [Figure 4 (iv)]. A similar linear interpolation correction scheme was used to compensate for extraordinarily bright pixels.

### 3.2. Real-time data reduction

The procedure developed in this research enables real-time pre-processing of hyperspectral data during data acquisition for reduction of the enormous Intensity hypercube, I(x,y,λ), into Score matrices, S(x,y), each representing a particular feature. This is achieved by multiplying the spectral emission of the target by an feature extraction operator predefined by experimentation. The simplest example of feature extraction is the pre-processing function of human vision. The spectral emission of a scene is multiplied by Red, Green and Blue (RGB) filters, the CIE 1931 colour matching functions [11] resulting three scalar values as score of features.

**Figure 5.**
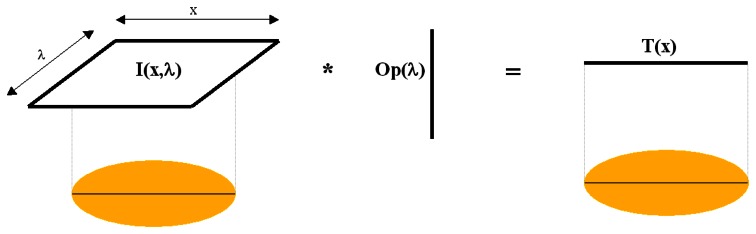
Conversion of 2-D spatial-spectral matrix, I(x,λ) into score matrix, T(x), as a result of a vector multiplication operator.

To perform real time feature extraction of hyperspectral images, the vector-to-scalar operator of the desired feature, Op(λ), must first be estimated by experimentation. An example of how this may be done is given further on in this paper. Once this has been done, while scanning the frame of an examined line, the 2-D (spatial vs spectral) Intensity matrix, I(x, λ), is real-time pre-processed into a score vector, T(x), by multiplying the spectrum of each pixel by Op(λ) (see Figure 5). By moving the object under the camera (in the y-direction) and grabbing the frames of subsequent lines, the scores matrix of features, T(x,y), is built. This matrix may be displayed while scanning as a pseudo-image, showing the distribution of the selected feature on the surface. Selected score matrices, which are substantially smaller in size than the corresponding hypercube (3-4 score image planes as opposed to >100 spectral planes in the hypercube) may then be saved for further analysis by usual image processing methods.

Optimal vector-to-scalar functions are usually determined by mathematical and statistical methods. For instance, Principal Component Analysis is frequently used in chemometrics. This analysis determines a set of independent base vectors, the first few of which optimally describe the variance of the sample population. The measured spectrum can then be transformed into the base vector-space [12]. For determining optimum data-reduction operators, spectra of samples have to be obtained where the examined feature of interest (e.g. moisture-content) is known. The optimal operator must then be determined by statistical analysis. The following section of this article shows an example of how to estimate an efficient operator for a given feature. In this case, the goal of the data reduction operator was to predict the moisture-content of carrot tissues during storage.

## 4. Experiments

Non-invasing monitoring of the impact of postharvest conditions on carrots can be an important tool in supply chain management. Spectrophotometric techniques have been adapted and evaluated with conventional chromatographic methods to test their feasibility for monitoring of compounds determining the quality of carrot during storage [13]. Investigating and modelling the behaviour of different tissues could be another interesting approach to describe the changes of carrot.

### Sample preparation

**Figure 6.**
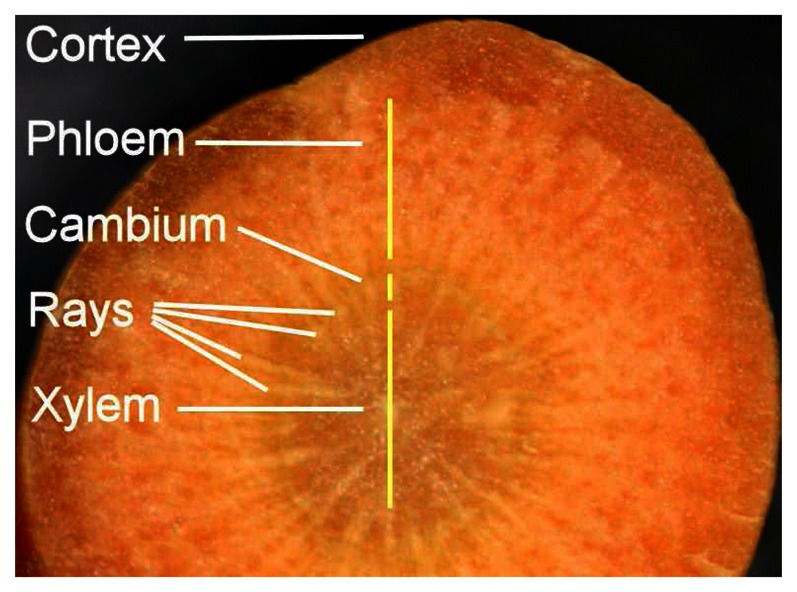
Cross section of carrot structure

**Figure 7.**
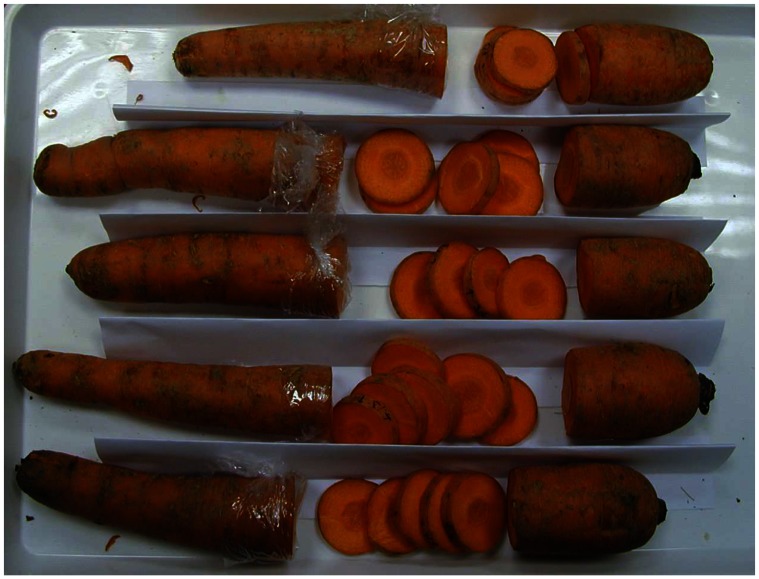
Film covered section (left) was cut each time to provide a fresh surface.

Carrot samples (Barbara cultivar) sourced from a local supplier were stored prior to analysis in a controlled atmosphere at a temperature of 4°C and a relative humidity of 90%. Cross-sectional slices were cut from the middle third of the carrot length, since this part is generally free from quality defects. Interior carrot structure is shown in Figure 6. At each time point studied, hyperspectral images of fresh-cut slices from the middle third of the carrot length were examined (Figure 7). Cross-sectioned carrot slices were measured rather than grated carrot, since grated carrot experienced rapid darkening by enzymatic oxidation of phenolic compounds. Re-measurement of a given slice resulted spectrally homogeneous whitening caused by drying of the surface layer; therefore it was necessary to measure fresh-cut surfaces at each time point. Plastic covering prevented the examined section from lengthwise drying since intact carrots lose moisture primarily in the radial direction during drying.

Carrot samples were dried to different moisture levels by storage in a drying chamber at 30 °C for 0, 90, 180, 270, 360, 450 min. Five carrots were used in the experiment: one slice was obtained from each of the 5 carrots at each time point, making five slice samples for each time point. Slices were weighed immediately after each hyperspectral measurement and were then dried completely for 24 hours drying in a chamber at 105 °C. Moisture content (% d.b.) was calculated by mass difference.

**Figure 8.**
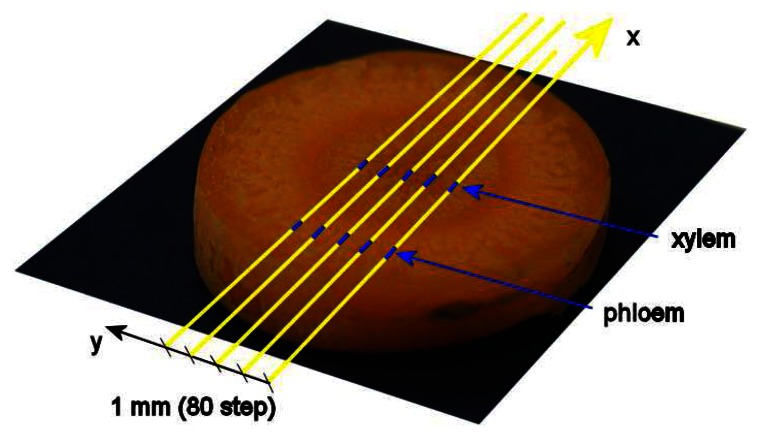
Areas of carrot tissue selected for analysis

Five hyperspectral line scans were obtained along the central region of selected carrot slices, as shown in Figure 8. The distance between scanning lines was 1 mm, and the average and variance of relative absorption was calculated along 3 mm of these lines at the sites of the phloem and xylem. Spectral data was limited to the range 950 – 1650 nm since the signal to noise ratio was unacceptably low beyond these limits. Due to temporal signal fluctuations, all spectra were normalized by dividing them by the mean value of the background signal obtained at respective time points.

## 5. Results

**Figure 9.**
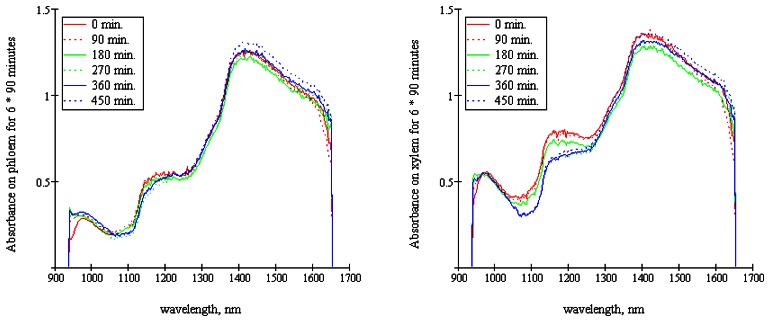
Average absorbance of carrot phloem and xylem (corrected by mean normalization) versus wavelength for different storage time intervals.

The average of 525 spectra was calculated for each of the drying times on both tissue types (Figure 9). Differences in spectral shape for the different time points studied were not readily visible, except for xylem absorbance in the 1050 – 1300 nm interval, which showed a trend of decreasing absorbance with increasing drying time. The observed decrease in absorption for longer drying times is probably related to water content, since liquid H_2_O exhibits an intermolecular stretch band at around 1200 nm [14].

Discriminant analysis proved that both the tissue types could be classified with very high scores along the whole measurement time (Table 1), and the time of storage could also be classified on both tissue-types (Table 2).

Finally, U-test or PLS analysis can be used to calculate the optimal wavelengths for discrimination. The linear combination of significant wavelengths can be divided by a wavelength that is invariant from the investigated property, to eliminate the changes of absorbance, caused by other circumstances, like illumination.

**Figure 10.**
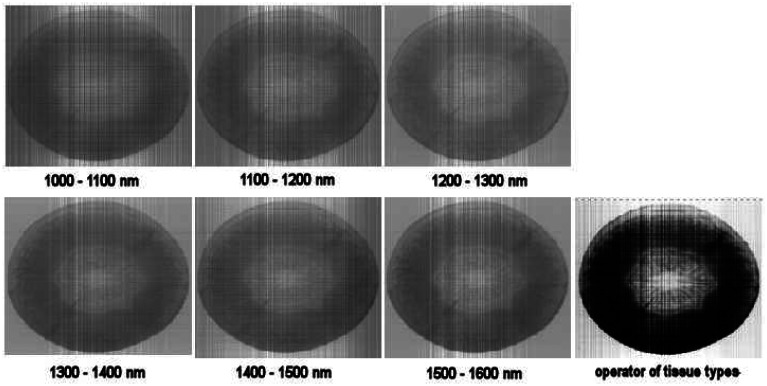
Pseudo-images of different ranges and one for enhancing xylem tissue type

**Figure 11.**
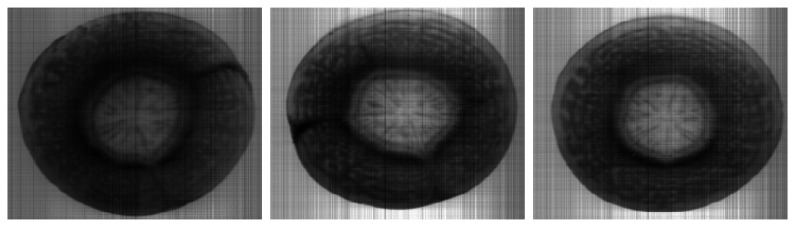
Pseudo-images to detect changes of xylem. Slices were cut after 0, 90 and 180 minutes storage time from a definite carrot.

Then the displayed pseudo-images of data-reduction operators can real-time display the distribution of examined property. Figure 10 illustrates the result of operator that classifies xylem tissue type on the base of significant wavelengths. The brightening of xylem tissues can be observed on the distribution of discriminant function that describes the change of xylem by time (Figure 11).

These pseudo-images of the measured object set can be processed later by the conventional image processing methods.

## 6. Conclusions

Measurement method was developed for insuring proper signal level of push-broom NIRHIS system and for reducing the data load of hyperspectral experiments by using sample-specific vector-to-scalar operators for real time feature extraction. The feasibility of this approach was tested for describing the changes of carrot tissues during storage. The method was able to distinguish the different behaviour of different tissues. Surfaces of food and raw materials can be investigated and tested by this method in preliminary measurements for using multispectral system for industrial tasks or quality control. Studying and modelling the behaviour of different tissues could be an interesting approach to describe more detailed changes in agricultural produce before harvest and during storage.

## Figures and Tables

**Table 1.** Classification results of DA grouping tissue types (xylem and phloem) for 2×630 spectra in calibration set and 2×2520 spectra in test set (data of each time intervals are contained)

			predicted group membership	
		original group	**xylem**	**phloem**
learning set: 2*630 spectra	count	**xylem**	630	0
		**phloem**	0	630
	%	**xylem**	**100.0**	0.0
		**phloem**	0.0	**100.0**
test set: 2*2520 spectra	count	**xylem**	2504	16
		**phloem**	20	2500
	%	**xylem**	**99.4**	0.6
		**phloem**	0.8	**99.2**

100.0% of selected original grouped cases (learning set) correctly classified.

99.3% of unselected original grouped cases (test set) correctly classified.

**Table 2.** Classification results of DA grouping different time intervals (0..5×90 min) for 6×210 spectra in calibration set and 6×840 spectra in test set (data of each tissue types are contained)

			predicted group membership					
		group	**0**	**90**	**180**	**270**	**360**	**450**
learning set: 6*210 spectra	%	**0**	**100.0**	0.0	0.0	0.0	0.0	0.0
		**90**	0.0	**98.6**	1.4	0.0	0.0	0.0
		**180**	0.0	4.3	**95.7**	0.0	0.0	0.0
		**270**	0.0	0.0	0.0	**81.0**	15.7	3.3
		**360**	0.0	0.0	0.0	5.2	**80.5**	14.3
		**450**	0.0	0.0	0.0	4.8	11.9	**83.3**
test set: 6*840 spectra	%	**0**	**98.1**	1.9	0.0	0.0	0.0	0.0
		**90**	0.1	**91.2**	8.7	0.0	0.0	0.0
		**180**	0.0	7.5	**92.5**	0.0	0.0	0.0
		**270**	0.0	0.0	0.0	**78.6**	16.9	4.5
		**360**	0.4	0.1	1.0	41.0	**41.9**	15.7
		**450**	0.0	0.0	0.0	38.9	26.2	**34.9**

89.8% of selected original grouped cases (learning set) correctly classified.

72.9% of unselected original grouped cases (test set) correctly classified.
